# Generalized pollination maintains disassortative pollen transfer in a distylous tropical shrub

**DOI:** 10.1093/aob/mcag199

**Published:** 2026-07-08

**Authors:** Luis Navarro

**Affiliations:** Departamento de Biología Vegetal y Ciencias del Suelo, Facultad de Biología, Universidade de Vigo, Campus As Lagoas-Marcosende, Vigo E-36310, Spain

**Keywords:** Distyly, functional redundancy, heterostyly, intermorph pollen transfer, pollination effectiveness

## Abstract

**Background and Aims:**

Distylous plants are expected to promote accurate intermorph pollen transfer through reciprocal positioning of sexual organs, yet many species are visited by taxonomically diverse pollinator assemblages. Whether generalized pollination compromises mating accuracy in such systems remains unclear.

**Methods:**

Single-visit experiments were conducted to quantify pollen transfer in the distylous Caribbean shrub *Morinda royoc* (Rubiaceae). Pollen-size dimorphism was used to distinguish morph-specific pollen and to estimate both total pollen deposition and the proportion of disassortative (reciprocal) pollen delivered by different visitor groups.

**Key Results:**

Multiple pollinator taxa, including butterflies, hummingbirds and bees, deposited predominantly intermorph pollen on stigmas. Although pollination effectiveness was unevenly distributed across the visitor assemblage and concentrated in a subset of species, the directionality of pollen flow remained consistently biased towards intermorph transfer across taxa. Variation in floral traits, including corolla tube dimensions, anther height, style length and stigmatic surface area, did not explain differences in the proportion of reciprocal pollen deposited.

**Conclusions:**

These findings indicate that reciprocal floral architecture can maintain mating accuracy under generalized pollination. Although quantitative contributions to pollination vary among visitors, the qualitative component of pollen transfer appears robust to differences in pollinator identity. Structural reciprocity might therefore stabilize reproductive processes in distylous systems exposed to variable pollinator assemblages.

## INTRODUCTION

Animal-mediated pollination underpins reproduction in most angiosperms, with community-level syntheses indicating that biotic pollination predominates across regions and is especially prevalent in the tropics (Ollerton *et al.*, 2011). This reliance on animal vectors creates strong scope for selection on floral traits that influence pollen placement and receipt, particularly in systems where successful fertilization depends on the transfer of compatible pollen between distinct mating types (Lloyd and Webb, 1992; Armbruster *et al.*, 2009).

Heterostyly is a widespread floral polymorphism that has evolved convergently across diverse angiosperm lineages. Populations comprise discrete floral morphs that differ in the reciprocal heights of stigmas and anthers (reciprocal herkogamy), often coupled with heteromorphic incompatibility (Darwin, 1877; Lloyd and Webb, 1992; Simón-Porcar *et al.*, 2024). Reviews emphasise that the defining structural components of heterostyly can enhance the proficiency of cross-pollination by promoting inter-morph (disassortative) pollen transfer while limiting pollen wastage via self- and intra-morph deposition (Barrett, 2019). Recent macroevolutionary analyses further support the idea that style-length polymorphisms tend to arise in lineages with floral features and pollination systems favouring precise pollen transfer, consistent with the pollination-precision hypothesis (Simón-Porcar *et al.*, 2024).

A central prediction of distyly is therefore functional: reciprocal placement of sex organs should increase the probability that pollen from one morph is deposited on stigmas of the opposite morph (‘legitimate’ or disassortative pollen transfer), thereby enhancing mating accuracy and reducing sexual interference between male and female functions (Darwin, 1877; Lloyd and Webb, 1992; Barrett *et al.*, 2000). Empirical evidence supports this prediction across multiple systems and approaches. Experimental manipulations in *Primula* demonstrated that heterostyly increases disassortative pollination and reduces sexual interference, directly linking floral reciprocity to measurable outcomes in pollen transfer and reproductive function (Keller *et al.*, 2014). Likewise, studies in distylous Rubiaceae have documented high levels of disassortative pollen transfer under animal pollination, including systems involving hummingbirds (Hernández and Ornelas, 2007). Comparative and experimental analyses in species exhibiting floral reciprocity, together with studies using artificial flowers further show that variation in reciprocal organ placement influences pollinator efficiency and intermorph pollen transfer (Ferrero *et al.*, 2011, 2025; Simón-Porcar *et al.*, 2014).

Despite these expectations, the efficiency of disassortative pollen transfer need not depend on pollination specialization defined solely by the number of floral visitor species (Armbruster, 2017). Taxonomic diversity of floral visitors and functional diversity in the way visitors handle flowers represent distinct dimensions of pollination generalization. A plant may be visited by several taxonomically unrelated species that contact anthers and stigmas in similar positions and therefore perform equivalent pollination functions. Conversely, a plant visited by few species may experience functionally divergent pollen transfer if those visitors differ in their approach, body position or the floral structures contacted during foraging. For heterostylous plants, the relevant distinction is therefore functional rather than taxonomic: visitors that consistently contact reciprocal sexual organs should promote more accurate intermorph pollen transfer, whereas visitors that differ in floral handling or pollen-placement sites might generate greater variation in pollen transfer among visits. Comparative analyses of morphometric data across numerous distylous Rubiaceae populations indicate that variation in reciprocal herkogamy (often quantified as inaccuracy) can influence the precision of intermorph pollen transfer across species and populations (Trevizan *et al.*, 2021). However, greater taxonomic and functional diversity among floral visitors might increase variation in pollen placement on pollinator bodies, because different visitor types often contact floral structures in distinct ways during foraging (Schemske and Horvitz, 1984; Herrera, 1987). Such variation in pollen placement might influence the consistency with which pollen from each morph is deposited on reciprocal stigmas in heterostylous species. Recent empirical work further illustrates how pollinator assemblage composition can influence the functional outcomes of heterostylous systems. In the distylous species *Linum narbonense*, variation in pollinator composition among populations was associated with marked differences in the quality of pollen received by the two floral morphs, with some visitor groups promoting effective intermorph pollination, whereas others were linked to reduced functional pollination success in one morph (Valverde, 2026). In generalized pollination systems involving multiple visitor species, interpretation of stigmatic pollen loads can be complicated further by the accumulation of pollen from successive visits, making it difficult to attribute deposited pollen to particular visitors (Adler and Irwin, 2006; Gómez *et al.*, 2025). Quantification of pollen deposition after single visits therefore provides the resolution necessary to disentangle visitor-specific effects on mating accuracy and to evaluate whether reciprocal floral architecture maintains disassortative pollen flow under diverse pollinator assemblages. These considerations reinforce the need for field-based studies that jointly quantify visitor assemblages and functional pollen transfer in heterostylous species, particularly in tropical settings where pollinator communities can include both insects and birds.

To address this need, in the present study I investigate a distylous Caribbean plant pollinated by a diverse assemblage of floral visitors, including hummingbirds, bees and butterflies. Building on measurements of floral morphology and nectar rewards, the composition of pollen loads deposited on stigmas was quantified and the degree of disassortative pollen transfer received by each morph estimated. I then tested whether variation in key floral traits, including corolla size and style length, predicts the proportion of intermorph pollen deposited on stigmas. By linking pollinator diversity to functional pollen transfer, this work evaluates whether generalized pollination can sustain efficient disassortative pollen transfer in a heterostylous system. Pollination effectiveness was evaluated by integrating a quantitative component, reflecting pollen delivery rate per flower, and a qualitative component, reflecting the proportion of reciprocal pollen transfer. This framework allows assessment not only of how much pollen is delivered by different floral visitors, but also of the extent to which that pollen contributes to reciprocal intermorph transfer in a distylous system. In this sense, the approach extends classical pollinator-importance frameworks by explicitly incorporating the quality of pollen transfer in terms of reciprocal pollen delivery.

## MATERIALS AND METHODS

### Study species

*Morinda royoc* L. (Rubiaceae) is a coastal shrub or small tree native to the Caribbean region, including Cuba and surrounding islands and continental coastal areas of the wider Caribbean Basin. It typically occurs in coastal scrub or thicket vegetation on limestone or coral-derived substrates and may grow on sandy or rocky coastal habitats (e.g. Espejel, 1987; Borhidi, 1996).

The species is distylous, producing two reciprocal floral morphs that differ in the relative positions of anthers and stigmas (short-styled vs. long-styled morphs). Flowers are arranged in dense, globose heads 7–10 mm in diameter and open early in the morning, remaining functional for a single day before withering by the end of the day. Each flower is enclosed basally by a fleshy, cup-shaped calyx that is tightly adnate to the inferior ovary, forming a distinct structural unit visible on the surface of the inflorescence. The tubular corolla emerges from within this calyx structure and bears five epipetalous stamens and a single style terminating in a bilobed stigma. The inferior ovary is bilocular, with two ovules (one per locule). Following fertilization, the ovaries of individual flowers within the inflorescence develop and fuse to form a fleshy syncarp. Each floral unit contributes one or two seeds, reflecting the bicarpellate structure of the ovary. The mature syncarp is yellow, juicy, and measures ∼2–3.5 cm in length and ∼2 cm in width. Fruits are present throughout much of the year and, in the study area, are consumed and dispersed by the Cuban rock iguana (*Cyclura nubila*) and frugivorous birds, such as *Spindalis zena* (Luis Navarro, unpublished data).

### Study site

Fieldwork was conducted in Guanahacabibes National Park, on the Guanahacabibes Peninsula (western Cuba), along a transect spanning the road/track corridor between Cabo San Antonio and María la Gorda. The peninsula is described as a low-lying limestone system with a marked seasonality in rainfall and a mosaic of coastal vegetation and inland limestone forests (Hernández *et al.*, 1994). The climate is tropical, with a pronounced dry season from November to April and a wet season from May to October. Mean annual precipitation is ∼1334 mm, and the mean annual minimum and maximum temperatures are 21.5 and 29.3 °C, respectively (Delgado *et al.*, 2005). The park includes extensive semi-deciduous limestone forest, mangrove-fringed margins in parts of the peninsula, and a variety of coastal plant communities on beaches, rocky ridges and terraces (Delgado *et al.*, 2005).

### Proportion of floral morphs and floral morphometrics

Floral morph frequencies and floral morphometrics were assessed using flowers collected in July 2007 from 57 randomly selected individuals along the Cabo San Antonio–María la Gorda transect in western Cuba. One flower per plant was sampled from each individual. Flowers were preserved in 70 % ethanol and transported to the laboratory at the University of Vigo (Spain) for analysis. In the laboratory, flowers were dissected, photographed on millimetric paper, and measured using ImageJ software (Schneider *et al.*, 2012). For each flower, the following traits were measured: (1) corolla length; (2) corolla tube width; (3) anther height for each of the five stamens; (4) style length; and (5) total stigmatic surface length, calculated as the sum of the lengths of the two stigma lobes. The degree of reciprocity between sexual organs of reciprocal floral morphs was quantified using the reciprocity index proposed by Sánchez *et al.* (2008, 2013), which compares differences in stigma–anther height across all potential intermorph combinations in the population. This index integrates both the magnitude and dispersion of stigma–anther separation and provides an unbiased quantitative estimate of reciprocity based on the empirical distributions of stigma and anther heights.

To assess whether pollen dimorphism was associated with heterostyly and could be used to distinguish pollen morphs under light microscopy, pollen size was measured from floral buds close to anthesis. One anther was collected from a floral bud about to open from each of five individuals per floral morph. Because pollen grains in this species are spheroidal, the longest diameter was used as a proxy for pollen size. In the laboratory, pollen grains were mounted on microscope slides and stained with a lactophenol–glycerin–Aniline Blue solution to enhance visibility. Measurements were performed using an ocular micrometer under a light microscope. For each floral morph, the longest diameter of 50 pollen grains was measured, including 10 grains per individual from each of the five sampled plants.

### Nectar production over 24 h

Nectar production over 24 h was assessed during the 2007 flowering season. Floral buds were bagged in the field on 12 plants (6 per floral morph) to prevent floral visitation. After 24 h, nectar volume was extracted from four flowers per plant using 10 µL Drummond microcapillary tubes. Sugar concentration (% w/w; °Brix) was measured using a handheld refractometer (Bellingham and Stanley, UK) adapted for small nectar volumes (0–50 % Brix scale). Sugar mass per flower (in milligrams) was calculated from nectar volume, and sugar concentration following standard conversion methods (Prŷs-Jones and Corbet, 1991). Energy content per flower (in kilojoules) was subsequently estimated using standard energetic equivalents for sugars (Prŷs-Jones and Corbet, 1991).

### Pollinator censuses and pollen deposition effectiveness

Pollinator censuses were conducted in natural populations of *M. royoc* in western Cuba during July 2006 and April 2007. Prior to observations, 62 individual plants were identified, and the number of open flowers per plant and floral morph were recorded. Observations were conducted from a distance of ∼2 m to minimize disturbance to floral visitors, particularly birds. During each census period, the identity of each floral visitor was recorded to the lowest taxonomic level possible, along with the number of flowers visited on each focal plant and the floral morph visited. Taxonomic identifications were subsequently verified using regional occurrence data from the Global Biodiversity Information Facility (GBIF; www.gbif.org). Floral visitors were assigned to functional groups according to the classification criteria described in Supplementary Data Methods S1. Total observation time amounted to 750 min in 2006 and 1020 min in 2007. Visitation frequency was quantified as the number of visits per flower and standardized to a 10 h observation period (07:00–17:00 h). This period corresponds to the daily interval during which floral visitors were observed to be active in natural field conditions. Standardization of the visitation frequency to a common 10 h period allowed comparison among visitor species independent of differences in total observation time.

To quantify pollen deposition effectiveness, newly opened flowers that had been protected from floral visitors by fine-mesh bags until the beginning of the daily censuses were identified and monitored during censuses. Immediately following a single visit, pistils were collected and preserved in 70 % ethanol for laboratory analysis, with the identity of the floral visitor recorded. Because pistils were collected immediately after a single visit, these counts were intended to estimate initial pollen deposition rather than subsequent pollen performance, compatibility responses, pollen-tube growth or fertilization success. In the laboratory, pistils were softened in sodium hydroxide (NaOH), stained with Aniline Blue, and examined under a Nikon epifluorescence microscope following established procedures for pollen visualization and quantification (Rojas-Nossa *et al.*, 2021). This procedure allowed the identification and counting of pollen grains deposited on each stigma and the discrimination between pollen originating from the same floral morph and pollen originating from the reciprocal morph based on differences in pollen size. This approach allowed quantification of both the total number of pollen grains deposited per visit and the proportion of disassortative pollen deposition per visit, defined as the fraction of pollen grains deposited on a stigma that originated from the reciprocal floral morph.

### Pollination effectiveness landscape in a distylous species

To evaluate the functional contribution of different floral visitors to pollination, a pollination effectiveness landscape framework was applied (Schupp *et al.*, 2010, 2017), integrating quantitative and qualitative aspects of pollen transfer. More generally, this approach follows the long-standing distinction between visitation frequency and per-visit pollinator effectiveness that underlies pollinator importance metrics (Herrera, 1987; Reynolds and Fenster, 2008). In the present study, this framework was adapted to a distylous system by explicitly incorporating the proportion of reciprocal pollen transfer as a measure of pollen-transfer quality. The quantitative component of pollination effectiveness was defined as the product of visitation frequency and stigmatic pollen deposition per visit. Visitation frequency estimates were derived from standardized pollinator census data. The pollen deposition component was calculated as the mean total pollen load deposited per visit for each floral visitor, pooling data from both floral morphs. This approach provides an estimate of the expected pollen delivery rate per flower per unit time. The qualitative aspect was characterized using reciprocal pollen deposition per visit, calculated as the mean proportion of reciprocal pollen deposited on stigmas by each floral visitor, pooling across floral morphs. In many distylous systems, pollen originating from the complementary morph represents the legitimate fraction of pollen transfer expected to contribute to compatible mating, whereas assortative pollen transfer is less likely to result in successful fertilization owing to spatial separation of sexual organs and incompatibility mechanisms commonly associated with heterostyly (Ganders, 1979; Barrett, 1990; Keller *et al.*, 2014). Accordingly, reciprocal pollen deposition per visit was used as a mechanistic proxy for pollen-transfer quality (QLC). This approach provides a biologically meaningful estimate of the effectiveness of floral visitors in promoting legitimate intermorph pollen transfer based on observed pollen deposition patterns, without requiring assumptions about the specific incompatibility mechanism operating in the species.

### Data analysis

All statistical analyses were conducted in R v.4.5.1 (R Core Team, 2025) and summary statistics are reported as the mean ± s.d.

Differences between floral morphs in floral traits, nectar production and pollen size were analysed using linear mixed-effects models, with floral morph included as a fixed effect and plant identity included as a random effect to account for the hierarchical structure of the data.

Differences among floral visitors and between floral morphs in total pollen deposition per visit were analysed using generalized linear models, with a negative binomial error distribution to account for overdispersion in count data. Differences in disassortative pollen deposition were analysed using generalized linear models with a binomial error distribution, modelling the number of pollen grains originating from the reciprocal morph relative to the total number of pollen grains deposited on each stigma. This approach accounts for the proportional nature of the response variable while retaining the information contained in the underlying pollen counts. In both cases, floral visitor identity, floral morph and their interaction were included as fixed effects.

*Post hoc* pairwise comparisons were conducted using estimated marginal means, with appropriate adjustments for multiple comparisons. Differences among floral visitors within each morph were evaluated using Tukey-adjusted contrasts.

## RESULTS

### Floral morph proportions, morphometrics and nectar production

A total of 57 individuals were sampled, comprising 28 long-styled and 29 short-styled plants, indicating an approximately balanced morph ratio within the study population.

Floral morphs differed markedly in the relative positions of sexual organs, consistent with reciprocal herkogamy (Fig. 1; Table 1). Anther height was significantly greater in short-styled flowers, whereas style length was significantly greater in long-styled flowers. In contrast, corolla length, corolla tube width and stigmatic surface length did not differ significantly between morphs (Table 1). The reciprocity index calculated following Sánchez *et al.* (2008) was 0.2488. Pollen size showed pronounced dimorphism, with short-styled flowers producing substantially larger pollen grains than long-styled flowers, as confirmed by linear mixed-effects modelling (Table 1).

**Fig. 1.**
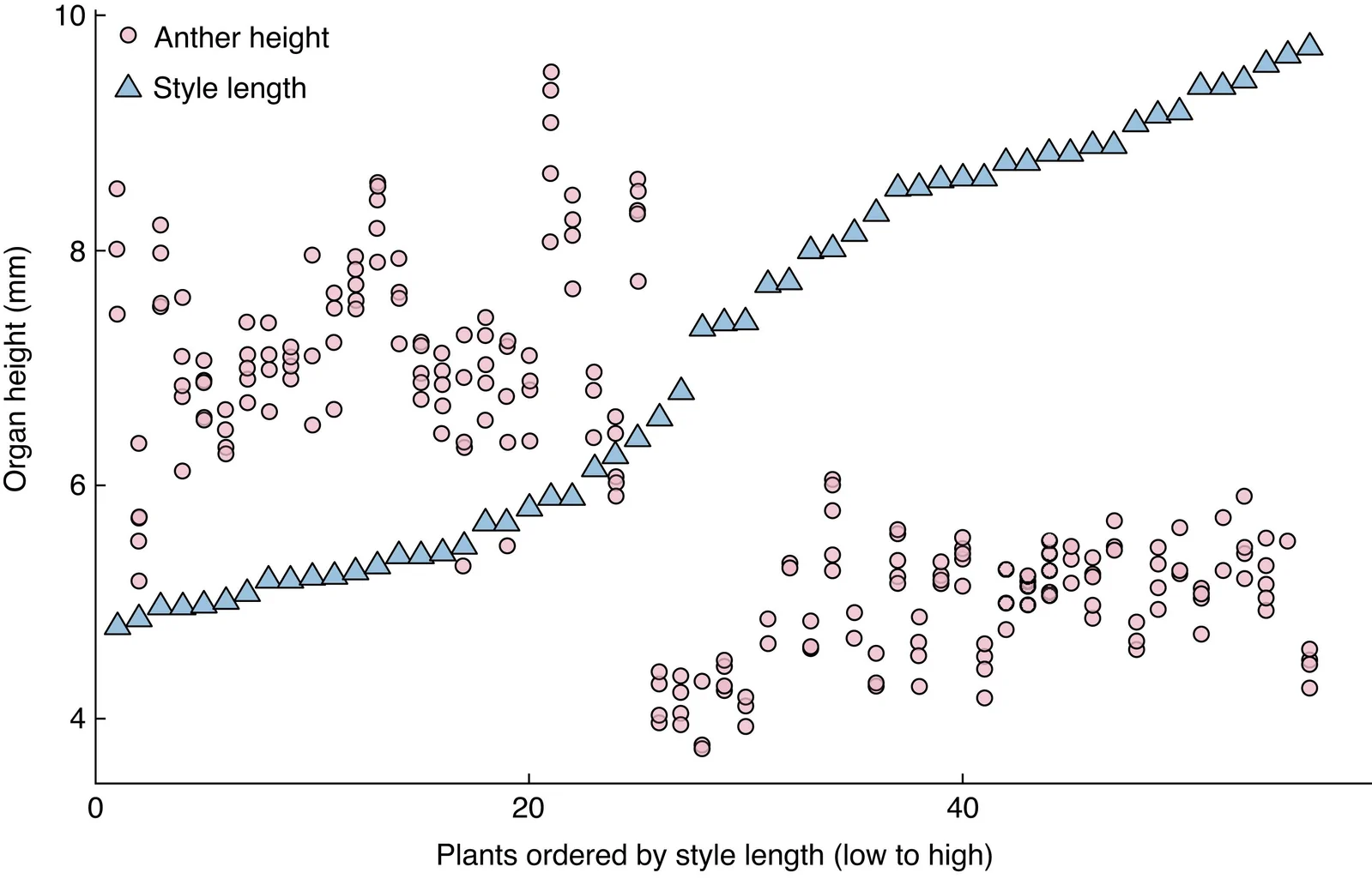
Reciprocal positioning of styles and stamens in distylous flowers of *Morinda royoc*. Style length (triangles) and individual anther heights (circles) are shown for plants ordered by increasing style length, illustrating reciprocal herkogamy between floral morphs.

**Table 1.** Floral morphological and nectar traits of long-styled and short-styled morphs of *Morinda royoc*.

Variable	L	S	*t*	d.f.	*P*-value (FDR-adjusted)
Corolla length (mm)	5.305 ± 0.753 (32)	5.262 ± 0.763 (24)	0.21	49.4	0.834
Corolla tube width (mm)	2.010 ± 0.222 (32)	1.966 ± 0.268 (24)	0.66	44.1	0.643
Style length (mm)	8.439 ± 0.912 (32)	5.377 ± 0.419 (24)	16.78	46.0	**<0.001**
Anther height (mm)	5.004 ± 0.540 (32)	7.231 ± 0.732 (24)	−12.57	40.6	**<0.001**
SSL (mm)	3.852 ± 0.753 (32)	4.230 ± 0.813 (24)	−1.78	47.5	0.136
Pollen size (µm)	37.24 ± 1.95 (5)	54.80 ± 2.66 (5)	−36.13	18.0	**<0.001**
Nectar volume (µL)	6.16 ± 2.02 (24)	5.83 ± 1.52 (24)	0.63	42.7	0.532
Nectar concentration (% w/w)	21.04 ± 2.49 (24)	19.21 ± 2.17 (24)	2.72	45.1	**0**.**037**
Nectar sugar (mg)	1.386 ± 0.430 (24)	1.204 ± 0.326 (24)	1.65	42.9	0.141

Values are the mean ± s.d. (sample size). Differences between morphs were assessed using Welch’s *t*-tests, except for anther height and pollen size, which were analysed using linear mixed-effects models with individual plant included as a random effect. The *P*-values were adjusted for multiple comparisons using the false discovery rate (FDR) procedure. Anther height corresponds to the mean value calculated from measurements of the five anthers per flower. Bold values indicate statistically significant differences between floral morphs. Abbreviations: L, long-styled morph; S, short-styled morph; SSL, stigma–stamen separation length.

Nectar volume did not differ significantly between floral morphs, but nectar sugar concentration was significantly higher in long-styled flowers. Consequently, sugar content and estimated energetic value per flower tended to be higher in long-styled flowers, although these differences were not statistically significant (Table 1).

### Floral traits and disassortative pollen flow

Both floral morphs received predominantly disassortative pollen loads, indicating extensive intermorph pollen transfer (Fig. 2). Long-styled flowers received on average 85.1 ± 19.0 % (mean ± s.d.) of pollen from the reciprocal morph, whereas short-styled flowers received 74.3 ± 32.5 %. None of the measured floral traits significantly predicted variation in disassortative pollen deposition. Corolla tube length, corolla tube width, style length and stigmatic surface length showed no significant association with the proportion of reciprocal pollen received (all *P* > 0.18; Supplementary Data Table S1). Fitted relationships between floral traits and disassortative pollen load were weak and showed no consistent directional trends (Supplementary Data Fig. S1).

**Fig. 2.**
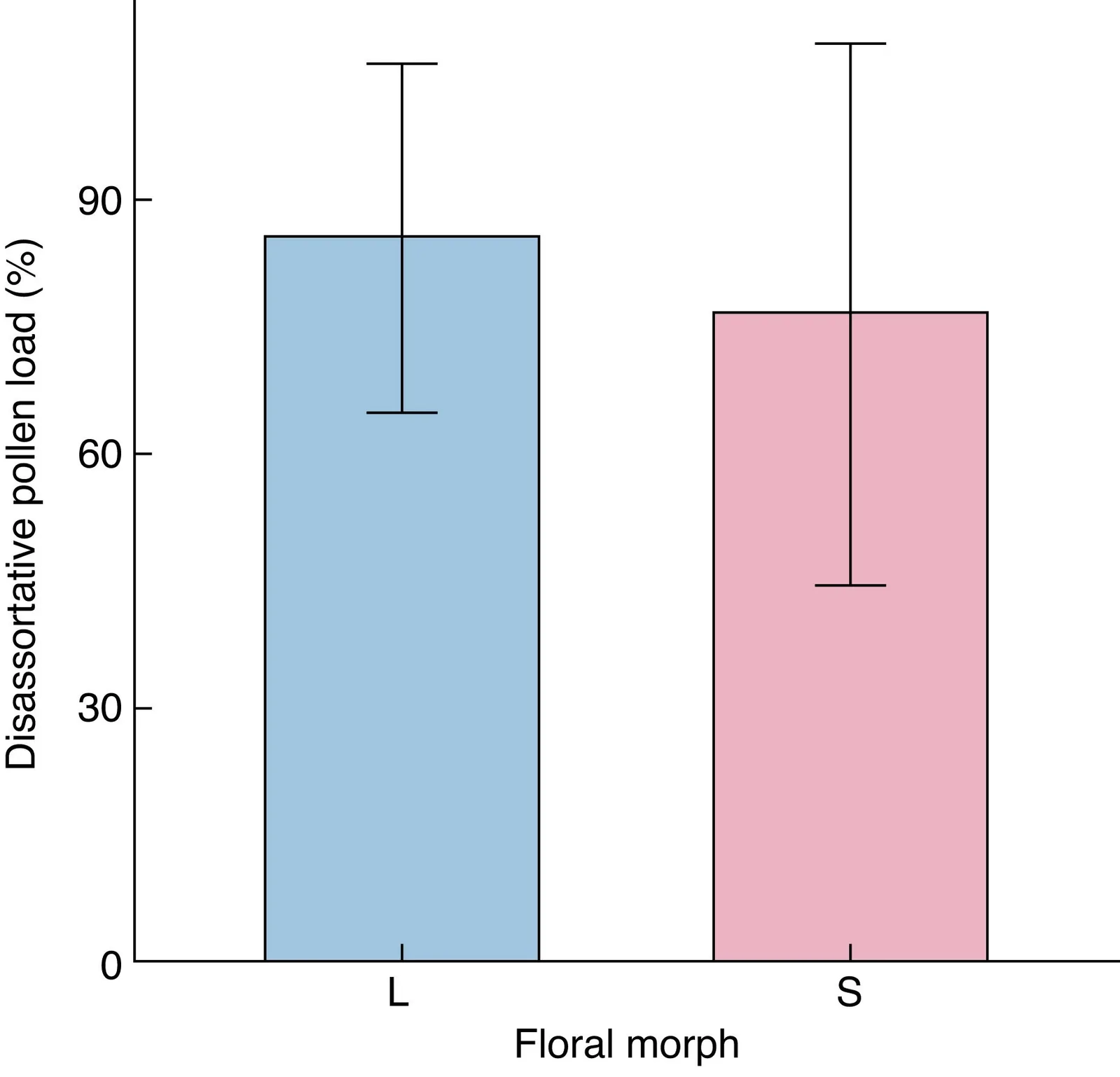
Disassortative pollen load in long-styled (L) and short-styled (S) flowers of *Morinda royoc*. Bars represent the mean ± s.d. percentage of pollen grains originating from the opposite floral morph.

### Pollinator censuses and pollen deposition effectiveness

A total of 592 flower visits by 13 visitor species representing multiple functional groups were recorded during 1770 min of observation (Table 2). Visitation frequency was strongly skewed towards a few dominant species representing distinct functional groups. The butterfly *Ascia monuste* accounted for 27.7 % of all visits, followed by the hummingbird *Mellisuga helenae* (23.1 %) and the large butterfly *Papilio andraemon* (18.9 %), whereas all other visitor species individually contributed <10 % of visits. When standardized to a 10 h observation period, visitation frequency ranged from 0.0020 visits per flower in the nectar robber passerine *Spindalis zena* to 0.1599 visits per flower in *A. monuste*, with intermediate values in *M. helenae* (0.1336) and *P. andraemon* (0.1092) (Table 2).

**Table 2.** Composition, visitation frequency and pollen deposition effectiveness of floral visitors to *Morinda royoc*.

Floral visitor	Family	Functional group	Visits (*n*)	Relative visitation frequency	Visits per flower (10 h)	*N*	Total pollen deposited per visit	Disassortative pollen deposition
*Ascia monuste*	Pieridae	Butterfly	164	0.277	0.1599 ± 0.2918	20	5.05 ± 1.99	0.9577 ± 0.1120
*Mellisuga helenae*	Trochilidae	Hummingbird	137	0.231	0.1336 ± 0.2718	20	10.5 ± 6.51	0.7842 ± 0.2367
*Papilio andraemon*	Papilionidae	Large butterfly	112	0.189	0.1092 ± 0.2939	20	6.8 ± 4.40	0.8701 ± 0.3047
*Ricordia ricordii*	Trochilidae	Hummingbird	43	0.073	0.0419 ± 0.1111	18	7 ± 4.85	0.6689 ± 0.3312
*Xylocopa cubaecola*	Apidae	Carpenter bee	39	0.066	0.0380 ± 0.1066	21	33.19 ± 15.95	0.7335 ± 0.2461
*Eurema dina*	Pieridae	Butterfly	24	0.041	0.0234 ± 0.1183	21	7.38 ± 4.44	0.8281 ± 0.2679
*Phoebis philea*	Pieridae	Large butterfly	23	0.039	0.0224 ± 0.0555	17	7.35 ± 3.87	0.9308 ± 0.1340
*Megachile apora*	Megachilidae	LPLB	14	0.024	0.0137 ± 0.0630	11	18.55 ± 7.02	0.7932 ± 0.2628
*Sphecodes genaroi*	Halictidae	SPLB	13	0.022	0.0127 ± 0.0585	11	7.64 ± 3.98	0.8337 ± 0.2130
*Halictus poeyi*	Halictidae	SPLB	12	0.020	0.0117 ± 0.0540	11	25.46 ± 10.26	0.5132 ± 0.3507
*Megachile singularis*	Megachilidae	LPLB	6	0.010	0.0059 ± 0.0312	NA	NA	NA
*Zethus cubensis*	Eumenidae	Wasp	3	0.005	0.0029 ± 0.0156	3	10.33 ± 6.81	0.4667 ± 0.5033
*Spindalis zena*	Spindalidae	NRP	2	0.003	0.0020 ± 0.0090	2	0 ± 0	0 ± 0

Floral visitors are listed with their taxonomic family, functional group, total number of visits and relative visitation frequency (proportion of total visits). Visits per flower (10 h) represents the expected number of visits received by a single flower over a standardized 10 h period (mean ± s.d.), calculated from visitation rates weighted by floral exposure. *N* indicates the number of pistils analysed following single visits. Total pollen deposited per visit represents the mean number of pollen grains deposited on stigmas (±s.d.). Disassortative pollen deposition represents the mean proportion of pollen originating from the reciprocal floral morph (±s.d.). Functional group assignment followed criteria described in the Supplementary Data Methods S1. Visitors are ordered by decreasing number of visits. Abbreviations: LPLB, long-proboscis large bee; NA, pollen deposition could not be quantified; NRP, nectar-robbing passerine; SPLB, short-proboscis large bee.

Pollen deposition effectiveness also varied substantially among visitor species. Total pollen deposition per visit ranged from 5.05 ± 1.99 grains in *A. monuste* to 33.19 ± 15.95 grains in *Xylocopa cubaecola*, with relatively high values also observed in the bees *Halictus poeyi* and *Megachile apora*. In contrast, butterflies such as *Phoebis philea* and *Eurema dina* deposited lower pollen loads. Most visitor species deposited predominantly disassortative pollen, with mean proportions exceeding 0.75 in several species, whereas lower values were observed in *H. poeyi* and *Zethus cubensis*. The effectiveness of pollen deposition could not be quantified for *Megachile singularis* or *S. zena* owing to the absence of analysable pistils following their visits.

### Visitor-specific differences in pollen deposition effectiveness

Floral visitors differed significantly in both the quantity and quality of pollen deposition (Fig. 3; Supplementary Data Tables S2 and S3). Total pollen deposition per visit varied substantially among visitor species and between floral morphs (Fig. 3A; Supplementary Data Table S2). The carpenter bee *Xylocopa cubaecola* deposited the largest pollen loads per visit and was the only visitor showing a significant difference between floral morphs, depositing more pollen on long-styled flowers than on short-styled ones. In contrast, most other visitor species deposited similar amounts of pollen on both floral morphs, despite differences in overall pollen deposition among species (Supplementary Data Table S2).

**Fig. 3.**
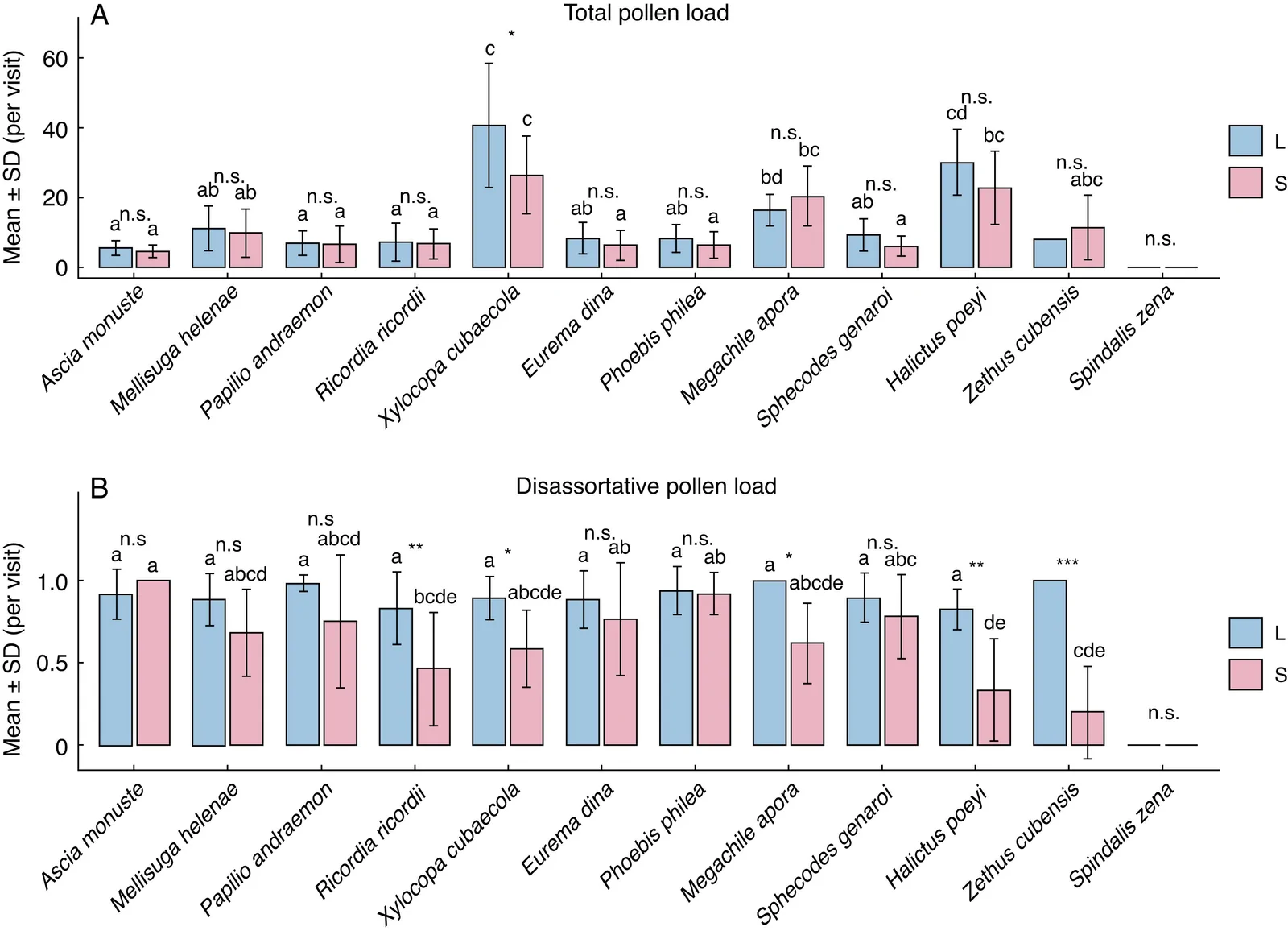
Quantitative and qualitative components of pollen transfer effectiveness for the floral visitor assemblage of *Morinda royoc*. (A) Mean (±s.d.) total pollen load deposited per visit. (B) Mean (±s.d.) proportion of disassortative pollen deposited per visit. Bars labelled L represent long-styled flowers, and bars labelled S represent short-styled flowers. Asterisks indicate significant differences between floral morphs within visitor species (*P < 0.05; **P < 0.01; ***P < 0.001; n.s., not significant). Different letters indicate significant differences among visitor species within each floral morph (Tukey-adjusted comparisons). Floral visitor species are arranged along the *x*-axis according to their visitation frequency recorded during pollinator censuses (see Table 2).

The proportion of disassortative pollen deposition per visit varied significantly among visitor species (Fig. 3B; Supplementary Data Table S3). Most visitor species deposited predominantly disassortative pollen, with mean proportions typically exceeding 0.6. Several species, including *Ascia monuste*, *Phoebis philea* and *Papilio andraemon*, showed particularly high proportions of disassortative pollen deposition. In contrast, lower mean proportions were observed in species such as *Halictus poeyi* and *Zethus cubensis*, particularly on short-styled flowers. Significant differences between floral morphs were detected in several visitor species, including *Ricordia ricordii*, *Xylocopa cubaecola*, *Megachile apora*, *Halictus poeyi* and *Zethus cubensis*, whereas no significant differences between morphs were observed for the remaining seven visitor species (Supplementary Data Table S3).

### Pollination effectiveness landscape

Integration of visitation frequency and pollen deposition effectiveness revealed marked differences among visitor species in both quantitative (QTC) and qualitative (QLC) components of pollination effectiveness (Fig. 4; Supplementary Data Table S4). Pollination effectiveness (PE), which integrates visitation frequency with both the quantity of pollen delivered per visit (QTC) and the proportion of disassortative pollen transfer (QLC), varied markedly among visitor species. Visitor species occupied distinct regions of the pollination effectiveness landscape, reflecting variation in visitation rates, pollen deposition per visit, and the proportion of disassortative pollen transfer. Overall pollination effectiveness was highest for species combining high visitation rates with effective pollen delivery. The hummingbird *Mellisuga helenae* and the carpenter bee *Xylocopa cubaecola* occupied regions of particularly high pollination effectiveness, reflecting their high QTC values, which combine relatively frequent visitation with substantial pollen deposition per visit. The butterfly *Ascia monuste* and the large butterfly *Papilio andraemon* also ranked among the most effective visitors, characterized by high proportions of disassortative pollen transfer (high QLC) and relatively high visitation rates. However, their QTC values were lower than those of the hummingbird and carpenter bee because each visit deposited fewer pollen grains on stigmas. In contrast, less frequent visitors, such as *Sphecodes genaroi* and *Zethus cubensis*, occupied regions of low pollination effectiveness, reflecting low visitation rates and/or reduced pollen-transfer quality. Pollination effectiveness could not be estimated for *Megachile singularis* and *Spindalis zena* because pollen deposition could not be quantified following their visits (see the Materials and Methods).

**Fig. 4.**
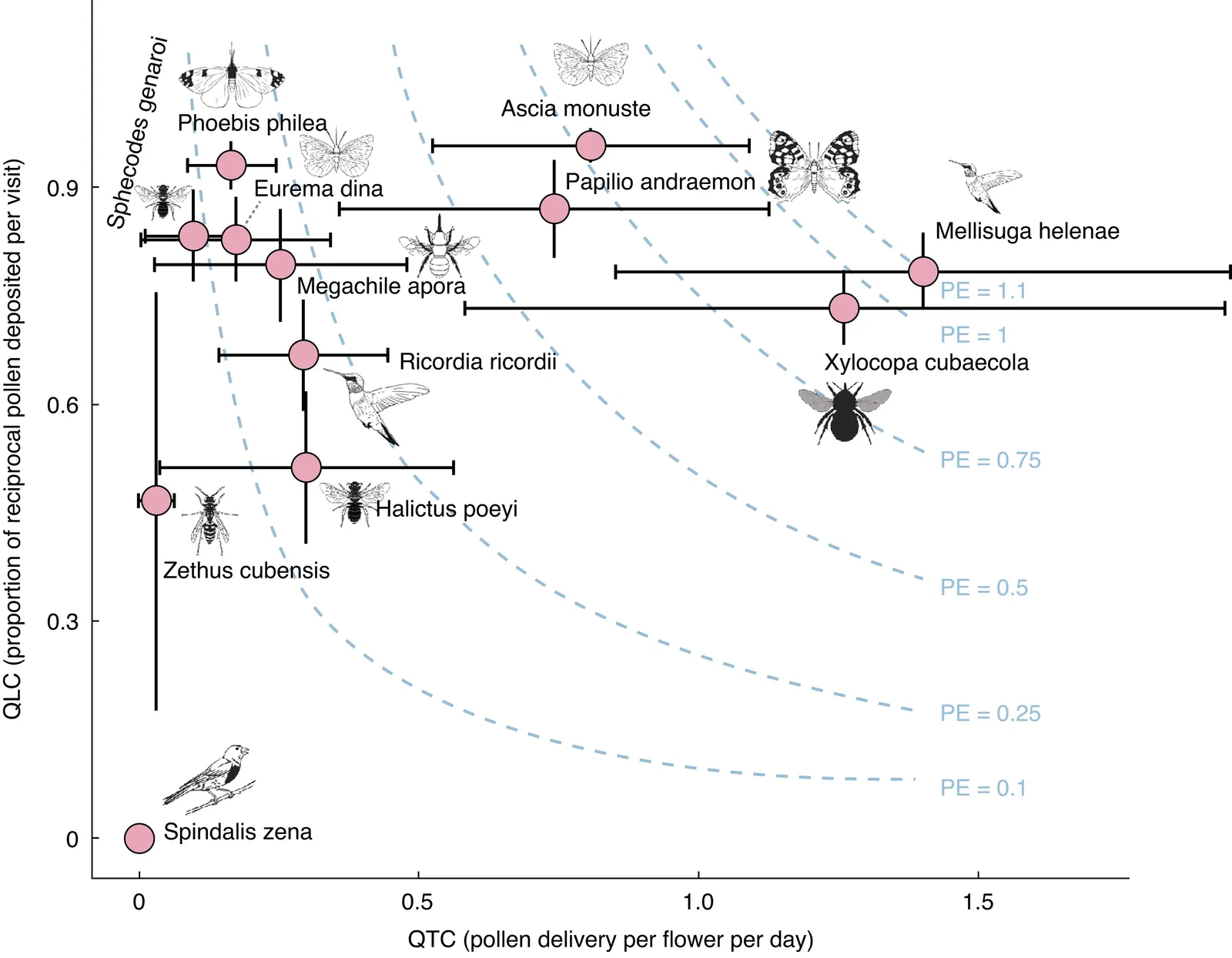
Pollination effectiveness landscape of floral visitors to *Morinda royoc*. QTC represents pollen delivery per flower over a standardized 10 h period, and QLC represents the proportion of reciprocal pollen deposited per visit. Points show species means ± s.e.; dashed lines represent isoclines of constant pollination effectiveness (PE = QTC × QLC). Floral visitors are ordered by visitation frequency (Table 2). See the Materials and Methods for calculation details. Floral visitor silhouettes were drawn by Divulgare (divulgare.net) under a Creative Commons licence (http://creativecommons.org/licenses/by-nc-sa/3.0).

## DISCUSSION

Distylous systems have classically been interpreted as adaptations that promote both disassortative mating and accurate intermorph pollen transfer under animal pollination (Darwin, 1877; Ganders, 1979; Barrett, 1990, 2019). The present study focuses specifically on the second of these functions. In *M. royoc*, the pronounced reciprocal herkogamy and clear pollen-size dimorphism between floral morphs provide both the mechanistic basis and the methodological resolution to quantify legitimate pollen transfer at the level of single visits. By using morph-diagnostic pollen size to partition stigmatic loads, the present study directly estimates the proportion of disassortative pollen transfer and links floral architecture to realized pollen flow. This approach follows a long tradition in heterostyly research, where pollen dimorphism has been used to infer intermorph versus intramorph transfer and to evaluate the functional consequences of reciprocity for mating patterns (Ganders, 1979; Barrett and Shore, 2008).

The results show that, despite a taxonomically diverse pollinator assemblage, multiple visitor groups deposit predominantly reciprocal pollen on stigmas. Legitimate pollen fractions remained high across butterflies, hummingbirds and bees, indicating that accurate intermorph transfer is not restricted to a single functional guild. Although the total amount of pollen deposited and visitation frequencies differed among visitors, the directionality of pollen flow was consistently biased towards disassortative transfer. These findings support the view that reciprocal floral architecture acts as a mechanical filter, promoting legitimate pollen exchange even under generalized pollination. Experimental and field studies in other distylous systems have likewise shown that reciprocal floral architecture can enhance intermorph pollen transfer and reduce sex organ interference (Hernández and Ornelas, 2007; Keller *et al.*, 2014), consistent with the patterns observed here.

Importantly, pollination effectiveness was not evenly distributed across the visitor assemblage. Effectiveness landscapes revealed strong functional differentiation, with a subset of species contributing disproportionately to total pollination success. In particular, the hummingbird *Mellisuga helenae* and the carpenter bee *Xylocopa cubaecola* combined relatively high pollen loads with high fractions of disassortative pollen deposition, placing them in the region of highest effectiveness. In contrast, some butterfly species deposited very high proportions of reciprocal pollen but contributed more modestly to total effectiveness owing to lower visitation rates or pollen loads. This decoupling between visitation frequency and pollen quality illustrates that generalized pollination in terms of visitor richness does not imply functional equivalence, a pattern consistent with studies showing that the reproductive consequences of plant–pollinator interactions are often dominated by a subset of particularly effective visitors within diverse assemblages (Gómez *et al.*, 2007, 2025).

The role of *Xylocopa cubaecola* is especially noteworthy. Carpenter bees are often associated with nectar robbing and reduced pollination efficiency in tubular flowers. In the same insular context, *X. cubaecola* has been documented acting as a nectar robber in *Duranta erecta* (Navarro and Medel, 2009). However, in *M. royoc*, this species functions as an effective pollinator, depositing substantial and largely reciprocal pollen loads. This contrast underscores the context dependence of pollinator function and suggests that floral architecture can modulate visitor behaviour and contact geometry in ways that transform potential robbers into legitimate pollen vectors. Reciprocal organ placement might therefore increase the likelihood that diverse visitors, once accessing floral rewards, contact sex organs in positions conducive to intermorph transfer.

Visitors with long bills or proboscides are morphologically well matched to the tubular corolla of *M. royoc*, facilitating deep nectar access while maintaining predictable contact with anthers and stigmas. Such morphological alignment is likely to enhance spatial segregation of pollen from different morphs on the body of the pollinator, increasing the probability of legitimate deposition. The importance of precise spatial matching between sex organs and pollinator contact zones has been emphasised in theoretical and empirical analyses of heterostyly (Lloyd and Webb, 1992; Armbruster *et al.*, 2009). However, the persistence of high disassortative fractions across multiple visitor types in the present study indicates that effective intermorph transfer is not exclusively dependent on a single morphological guild. This contrasts with recent evidence from other distylous systems, where pollinator composition can strongly influence the functional quality of pollen receipt by different floral morphs. For example, Valverde (2026) showed that in *Linum narbonense*, a distylous species with a bowl-shaped corolla, the proportion of compatible pollen reaching stigmas varied with the relative contribution of different pollinator groups, leading to morph-specific differences in pollination quality across populations. Differences in floral architecture might explain this contrast in part, because open, bowl-shaped flowers allow multiple approaches and contact positions by visitors, whereas the tubular corolla of *M. royoc* constrains the interaction between floral visitors and reproductive organs, potentially promoting more consistent pollen placement (Lloyd and Webb, 1992; Armbruster *et al.*, 2009). In the present system, once a threshold of functional pollinator fit is met, reciprocal herkogamy appears sufficient to promote consistent directional pollen transfer. This structural constraint might therefore buffer the qualitative component of pollination against variation in pollinator identity within generalized visitor assemblages.

Interestingly, measured floral traits, such as corolla dimensions, tube length and stigma area, did not significantly predict the proportion of reciprocal pollen deposited. This suggests that, within the observed range of morphological variation, structural reciprocity at the population level might override finer-scale variation in floral dimensions. The realized fraction of disassortative pollen transfer might thus depend more strongly on visitor behaviour, body positioning and grooming patterns than on subtle differences in floral morphology. These findings reinforce the view that floral architecture and pollinator behaviour must be considered jointly to understand mating outcomes in heterostylous systems (Armbruster *et al.*, 2009; Keller *et al.*, 2014; Armbruster, 2017).

The presence of clear pollen-size dimorphism and reciprocal herkogamy in *M. royoc* further supports the interpretation of this species as a canonical distylous system rather than a functionally dioecious one, as has been suggested for some taxa historically placed within *Morinda* (e.g. Razafimandimbison and Bremer, 2011; Liu *et al.*, 2012).

Although heteromorphic incompatibility was not tested directly in the present study, the use of morph-diagnostic pollen size allowed estimation of the legitimate (intermorph) fraction of pollen transfer, which represents the mechanistic prerequisite for compatible mating in distylous systems. The incompatibility system of *M. royoc* remains uncharacterized, and therefore the present results should be interpreted as estimates of intermorph pollen transfer rather than direct measures of compatible pollen performance. Nevertheless, the presence of pronounced reciprocal herkogamy and pollen-size dimorphism are consistent with the classical distylous syndrome described in Rubiaceae (Ganders, 1979). The presence of clear pollen-size dimorphism and reciprocal herkogamy in *M. royoc* further support interpretation of this species as a canonical distylous system rather than a functionally dioecious one, as has been suggested for some taxa historically placed within *Morinda* (e.g. Razafimandimbison and Bremer, 2011; Liu *et al.*, 2012).

Taken together, the results indicate that accurate intermorph pollen transfer in *M. royoc* is maintained under a generalized pollinator assemblage. Although total pollination effectiveness is concentrated in a subset of visitors, the qualitative component of pollination (the directionality of pollen flow) remains consistently disassortative across taxa. This pattern suggests a degree of functional redundancy in the maintenance of legitimate pollen transfer. Although the present study did not test resilience experimentally through pollinator exclusion, the distribution of disassortative transfer among visitor groups implies that highly reciprocal distylous systems might be buffered against moderate fluctuations in pollinator composition. More broadly, these findings highlight how structural traits, such as reciprocal herkogamy, can stabilize mating patterns under ecological variability. Rather than requiring strict pollinator specialization, distylous floral architecture might enable robust intermorph pollen exchange across diverse visitor assemblages. By linking floral reciprocity, pollinator effectiveness and realized pollen transfer at the level of single visits, this study provides mechanistic insight into how heterostylous reproductive systems maintain mating accuracy in generalized pollination environments.

## Supplementary Material

mcag199_Supplementary_Data

## Data Availability

Data and R scripts supporting the results of this study will be available upon request.

## SUPPLEMENTARY DATA

Supplementary data are available at *Annals of Botany* online and consist of the following. Methods S1: functional group classification of floral visitors. Table S1: effects of floral morphological traits on disassortative pollen deposition. Table S2: total pollen deposition per visit by floral visitors. Table S3: disassortative pollen deposition by floral visitors. Table S4: pollination effectiveness components. Figure S1: relationship between floral morphology and disassortative pollen load.
