# Supplementary material for: Generalized pollination maintains disassortative pollen transfer in a distylous tropical shrub
Source: Ann Bot. 2026 Jul 8;138(5):1682–92. doi: 10.1093/aob/mcag199 (PMC13714995; doi:10.1093/aob/mcag199)
Supplement: Supplementary file 1 [file mcag199_supplementary_data.docx]

**Supplementary Methods S1. Functional group classification of floral visitors**

Definition and rationale of functional groups. Floral visitors were classified into functional groups based on shared morphological and behavioural traits expected to influence their interaction with flowers and their effectiveness as pollen vectors. In diverse pollination systems, multiple pollinator species often interact with flowers in broadly similar ways and can therefore exert comparable ecological and selective effects on plants. For this reason, pollinators are frequently grouped into functional categories defined by convergent traits that determine how they access floral rewards and contact reproductive structures (Fenster et al., 2004; Gómez et al., 2015; Gómez et al., 2016). Functional groups thus represent ecologically meaningful interaction units in generalized pollination systems, allowing comparisons among visitors that differ in their potential to contact reproductive organs, transfer pollen between floral morphs, and move pollen across spatial scales.

In heterostylous species such as *Morinda royoc*, functional grouping is particularly relevant because effective disassortative pollen transfer depends on the spatial positioning of pollen on the pollinator’s body and on movement between individuals bearing reciprocal floral morphs. Traits such as body size, proboscis length relative to corolla depth and foraging behaviour influence both pollen placement and carryover, as well as the likelihood of movement between plants.

Functional groups were assigned from direct field observations of how each visitor species interacted with *M. royoc* flowers. The distinction between medium-sized and large visitors, as well as between bees with relatively short or long proboscides, was based on their size and morphology relative to the floral tube, together with their mode of nectar access and the resulting contact with the reproductive organs. Thus, the groups reflect functional differences in flower–visitor interactions rather than predefined morphometric thresholds.

**Functional group definitions**

**Hummingbird.** Includes nectar-feeding birds (Trochilidae) that access nectar legitimately through the corolla opening. Their hovering or perching behavior and frequent contact with anthers and stigmas make them effective pollen vectors. Their high mobility allows pollen transfer over relatively long distances.

**Butterfly.** Includes medium-sized Lepidoptera (e.g. Pieridae such as *Ascia monuste* and *Eurema dina*) that forage while perched and access nectar using a proboscis inserted through the corolla opening. These visitors typically contact reproductive structures with the head or proboscis base and may transfer pollen between nearby individuals.

**Large butterfly (LB).** Includes larger butterfly species (e.g. Papilionidae such as *Papilio andraemon*, and large Pieridae such as *Phoebis philea*) characterized by greater body size and wingspan. Their larger size may alter patterns of contact with floral reproductive organs, and their greater displacement capacity may facilitate pollen transfer over larger spatial scales.

**Carpenter bee.** Includes large-bodied bees of the genus *Xylocopa*, which in this plant species forage legitimately by inserting their head and often part of the thorax into the corolla tube. Their large body size and robust morphology promote extensive and consistent contact with both anthers and stigmas. Carpenter bees were distinguished from other large bees because their head-first insertion into the corolla and relatively shorter glossae relative to body size result in predictable pollen placement on specific body regions (primarily the head and dorsal thorax). This behaviour and morphology are expected to promote efficient pollen pickup and deposition, particularly in heterostylous systems where spatial correspondence between pollen placement and stigma position is critical.

**Long proboscis large bee (LPLB).** Includes large-bodied bees possessing relatively long proboscides (e.g. certain Megachilidae), enabling nectar access from tubular flowers without fully inserting the head or thorax into the corolla. These bees can obtain nectar while maintaining partial contact with reproductive organs, often resulting in pollen placement on the head or proboscis base. Their morphology and foraging behaviour may therefore produce pollen transfer patterns distinct from those of bees that must insert more of the body into the flower.

**Short proboscis large bee (SPLB).** Includes large-bodied bees with shorter proboscides relative to corolla depth. These visitors typically need to insert the head or part of the thorax into the corolla tube to access nectar, resulting in more extensive contact with reproductive structures. Such contact may promote pollen deposition on broader body regions and influence patterns of pollen carryover and deposition.

**Wasp.** Includes nectar-feeding wasps that access nectar legitimately through the corolla opening. Although capable of contacting reproductive organs, wasps typically lack the dense branched body hairs (plumose setae) characteristic of bees, which facilitate pollen adhesion and retention. As a result, pollen grains may adhere less efficiently to their body surface and may be more easily lost during flight or grooming. Consequently, wasps are generally considered less efficient pollen vectors than bees of comparable size, although they may still contribute to pollen transfer through incidental contact during nectar foraging.

**Nectar robber passerine (NRP).** Includes nectar-robbing birds such as *Coereba flaveola* or *Spindalis zena* that obtain nectar by piercing or accessing the corolla outside the floral opening. This behaviour often reduces direct contact with reproductive structures and may limit pollen transfer, although secondary pollen transfer may occur during incidental contact.

**References**

Fenster CB, Armbruster WS, Wilson P, Dudash MR, Thomson JD. 2004. Pollination syndromes and floral specialization. *Annual Review of Ecology, Evolution, and Systematics* 35: 375–403.

Gómez JM, Perfectti F, Lorite J, Klingenberg CP, Torices R. 2015. Evolution of pollination niches in a generalist plant clade. *New Phytologist* 205: 440–453.

Gómez JM, Torices R, Lorite J, Klingenberg CP, Perfectti F. 2016. The role of pollinators in the evolution of corolla shape variation, disparity and integration in a highly diversified plant family with a conserved floral bauplan. *Annals of Botany* 117: 889–904.

**Table S1.** Effects of floral morphological traits on the proportion of disassortative pollen deposition in *Morinda royoc*. Results from a binomial generalized linear model testing whether corolla length, corolla tube width, style length, and stigmatic surface length predict the proportion of pollen received from the reciprocal floral morph. Estimates are shown on the logit scale.

| **Term** | **estimate** | **std. error** | **z-valuec** | **p value** |
| --- | --- | --- | --- | --- |
| (Intercept) | 7.399 | 2.914 | 2.54 | 0.015 |
| Corolla length | -0.382 | 0.3728 | -1.02 | 0.312 |
| Corolla tube width | -1.249 | 1.177 | -1.06 | 0.296 |
| Style length | 0.143 | 0.146 | 0.98 | 0.333 |
| Stigmatic surface length | -0.496 | 0.295 | -1.68 | 0.101 |

**Table S2.** Total pollen deposition per visit by each floral visitor species to long-styled (L) and short-styled (S) flowers of *Morinda royoc*. Values are mean ± SD, and N indicates the number of pistils analysed following single visits. P (L vs. S) indicates the probability value from Welch’s t-tests comparing pollen deposition between floral morphs within each visitor species. Significance codes: n.s., not significant (P ≥ 0.05); ***** P < 0.05. Tests involving very small sample sizes should be interpreted with caution.

| **Floral visitor** | **Morph** | **N** | **Mean ± SD** | **P (L vs S)** | **Significance** |
| --- | --- | --- | --- | --- | --- |
| *Ascia monuste* | L | 10 | 5.5 ± 2.12 | 0.508 | n.s. |
| *Ascia monuste* | S | 10 | 4.6 ± 1.84 |  |  |
| *Mellisuga helenae* | L | 10 | 11.2 ± 6.39 | 0.559 | n.s. |
| *Mellisuga helenae* | S | 10 | 9.8 ± 6.89 |  |  |
| *Papilio andraemon* | L | 10 | 6.9 ± 3.6 | 0.906 | n.s. |
| *Papilio andraemon* | S | 10 | 6.7 ± 5.27 |  |  |
| *Ricordia ricordii* | L | 10 | 7.2 ± 5.53 | 0.806 | n.s. |
| *Ricordia ricordii* | S | 8 | 6.75 ± 4.2 |  |  |
| *Xylocopa cubaecola* | L | 10 | 40.6 ± 17.72 | **0.027** | ***** |
| *Xylocopa cubaecola* | S | 11 | 26.45 ± 11.03 |  |  |
| *Eurema dina* | L | 11 | 8.36 ± 4.48 | 0.241 | n.s. |
| *Eurema dina* | S | 10 | 6.3 ± 4.37 |  |  |
| *Phoebis philea* | L | 9 | 8.22 ± 4.02 | 0.344 | n.s. |
| *Phoebis philea* | S | 8 | 6.38 ± 3.7 |  |  |
| *Megachile apora* | L | 5 | 16.4 ± 4.56 | 0.450 | n.s. |
| *Megachile apora* | S | 6 | 20.33 ± 8.57 |  |  |
| *Sphecodes genaroi* | L | 5 | 9.4 ± 4.67 | 0.202 | n.s. |
| *Sphecodes genaroi* | S | 6 | 6.17 ± 2.93 |  |  |
| *Halictus poeyi* | L | 4 | 30.0 ± 9.49 | 0.335 | n.s. |
| *Halictus poeyi* | S | 7 | 22.86 ± 10.43 |  |  |
| *Zethus cubensis* | L | 1 | 8.0 ± 0.0 | 0.574 | n.s. |
| *Zethus cubensis* | S | 2 | 11.5 ± 9.19 |  |  |
| *Spindalis zena* | L | 1 | 0.0 ± 0.0 | 1 | n.s. |
| *Spindalis zena* | S | 1 | 0.0 ± 0.0 |  |  |

**Table S3.** Disassortative pollen deposition by floral visitors to *Morinda royoc*. Values represent the mean proportion of pollen deposited per visit that originated from the reciprocal floral morph (mean ± SD). N indicates the number of pistils analysed following single visits. P (L vs. S) indicates the significance of differences between long-styled (L) and short-styled (S) flowers for each visitor species (Welch’s t-tests). Significance levels: n.s., P ≥ 0.05; * P < 0.05; ** P < 0.01; *** P < 0.001. Tests involving very small sample sizes should be interpreted with caution.

| **Floral visitor** | **Morph** | **N** | **Mean ± SD** | **P (L vs S)** | **Significance** |
| --- | --- | --- | --- | --- | --- |
| *Ascia monuste* | L | 10 | 0.915 ± 0.15 | 0.526 | n.s. |
| *Ascia monuste* | S | 10 | 1.0 ± 0.0 |  |  |
| *Mellisuga helenae* | L | 10 | 0.887 ± 0.157 | 0.186 | n.s. |
| *Mellisuga helenae* | S | 10 | 0.682 ± 0.265 |  |  |
| *Papilio andraemon* | L | 10 | 0.985 ± 0.049 | 0.096 | n.s. |
| *Papilio andraemon* | S | 10 | 0.756 ± 0.406 |  |  |
| *Ricordia ricordii* | L | 10 | 0.833 ± 0.221 | **<0.001** | ****** |
| *Ricordia ricordii* | S | 8 | 0.464 ± 0.343 |  |  |
| *Xylocopa cubaecola* | L | 10 | 0.896 ± 0.13 | **0.029** | ***** |
| *Xylocopa cubaecola* | S | 11 | 0.586 ± 0.235 |  |  |
| *Eurema dina* | L | 11 | 0.885 ± 0.176 | 0.536 | n.s. |
| *Eurema dina* | S | 10 | 0.766 ± 0.342 |  |  |
| *Phoebis philea* | L | 9 | 0.94 ± 0.145 | 0.780 | n.s. |
| *Phoebis philea* | S | 8 | 0.92 ± 0.129 |  |  |
| *Megachile apora* | L | 5 | 1.0 ± 0.0 | **0.039** | ***** |
| *Megachile apora* | S | 6 | 0.621 ± 0.244 |  |  |
| *Sphecodes genaroi* | L | 5 | 0.896 ± 0.152 | 0.697 | n.s. |
| *Sphecodes genaroi* | S | 6 | 0.782 ± 0.256 |  |  |
| *Halictus poeyi* | L | 4 | 0.825 ± 0.123 | **<0.001** | ****** |
| *Halictus poeyi* | S | 7 | 0.335 ± 0.31 |  |  |
| *Zethus cubensis* | L | 1 | 1.0 ± 0.0 | **<0.001** | ******* |
| *Zethus cubensis* | S | 2 | 0.2 ± 0.283 |  |  |
| *Spindalis zena* | L | 1 | 0.0 ± 0.0 | 1 | n.s. |
| *Spindalis zena* | S | 1 | 0.0 ± 0.0 |  |  |

**Table S4.** Quantitative (QTC), qualitative (QLC), and overall pollination effectiveness (PE) components for floral visitors of *Morinda royoc*. Visits per flower (10 h) represents the expected number of visits received by a single flower over a standardized 10-h period, calculated from visitation rates weighted by floral exposure. Pollen deposited per visit represents the mean number of pollen grains deposited on stigmas following single visits. QTC (quantitative component) represents pollen delivery per flower over a standardized 10-h period, calculated as visitation frequency multiplied by pollen deposition per visit. QLC (qualitative component) represents the mean proportion of pollen deposited that originated from the reciprocal floral morph. Pollination effectiveness (PE) integrates both components and was calculated as PE = QTC × QLC. NA indicates that pollen deposition could not be quantified; consequently, QTC, QLC and PE could not be calculated.

| **Floral visitor** | **Visits per flower (10 h)** | **Pollen deposited per visit (mean)** | **Disassortative pollen proportion (QLC)** | **QTC** | **QLC** | **Pollination effectiveness (PE)** |
| --- | --- | --- | --- | --- | --- | --- |
| *Mellisuga helenae* | 0.134 | 10.5 | 0.784 | 1.403 | 0.784 | 1.1 |
| *Xylocopa cubaecola* | 0.038 | 33.19 | 0.733 | 1.262 | 0.733 | 0.926 |
| *Ascia monuste* | 0.16 | 5.05 | 0.958 | 0.808 | 0.958 | 0.773 |
| *Papilio andraemon* | 0.109 | 6.8 | 0.87 | 0.743 | 0.87 | 0.646 |
| *Megachile apora* | 0.014 | 18.55 | 0.793 | 0.253 | 0.793 | 0.201 |
| *Ricordia ricordii* | 0.042 | 7 | 0.669 | 0.294 | 0.669 | 0.196 |
| *Phoebis philea* | 0.022 | 7.35 | 0.931 | 0.165 | 0.931 | 0.153 |
| *Halictus poeyi* | 0.012 | 25.45 | 0.513 | 0.298 | 0.513 | 0.153 |
| *Eurema dina* | 0.023 | 7.38 | 0.828 | 0.173 | 0.828 | 0.143 |
| *Sphecodes genaroi* | 0.013 | 7.64 | 0.834 | 0.097 | 0.834 | 0.081 |
| *Zethus cubensis* | 0.003 | 10.33 | 0.467 | 0.03 | 0.467 | 0.014 |
| *Spindalis zena* | 0.002 | 0 | 0 | 0 | 0 | 0 |
| *Megachile singularis* | 0.006 | NA | NA | NA | NA | NA |

**Figure S1.** Relationship between floral morphology and disassortative pollen load in *Morinda royoc*. Disassortative pollen load (%) in individual flowers plotted against (a) corolla length, (b) corolla tube width, (c) style length, and (d) stigmatic surface length. Symbols indicate floral morph (long-styled, L; short-styled, S). Lines show fitted values from quasi-binomial generalized linear models, with shaded bands representing 95% confidence intervals.
